# Single-cell analysis of the cellular landscape of vulvar melanoma provides new insight for immunotherapy administration

**DOI:** 10.1186/s12885-024-11839-0

**Published:** 2024-01-17

**Authors:** Xinqi Wang, Jiahui Li, Yifei Li, Mingyi Lv, Xue Dong, Zhenxin Fan, Tao Guo

**Affiliations:** 1 Key Laboratory of Bioresources and Eco-environment (Ministry of Education), College of Life Sciences, Sichuan University, 610065 Chengdu, Sichuan China; 2 Key Laboratory of Birth Defects and Related Diseases of Women and Children of MOE, Department of Pediatrics, West China Second University Hospital, Sichuan University, 610041 Chengdu, Sichuan China; 3 Ambulatory surgery Department, West China Second Hospital, Sichuan University, 610041 Chengdu, Sichuan China; 4 Department of Gynecology and Obstetrics, West China Second University Hospital, Sichuan University, 610041 Chengdu, Sichuan China

**Keywords:** Vulvar melanoma, scRNA-seq, Immune microenvironment, Immune cells; Case report

## Abstract

**Background:**

Vulvar and vaginal melanoma (VuM & VaM) is a rare gynecologic malignancy with high mortality but low effectiveness to checkpoint immunotherapy compared to cutaneous melanoma. This article aims to elucidate the role of the disordered immune microenvironment in cancer progression in VuM.

**Methods:**

At first, this article applied single-cell RNA sequencing (scRNA-seq) to the VuM obtained from a 68-year-old female patient, and constructed a single-cell atlas of VuM consist of 12,243 single cells. Then this article explores the genomic complexity and core signal channel in VuM microenvironment.

**Results:**

This article provides new insights about the pathogenesis of VuM based on single-cell resolution data. It was found that the activation of CD8^+^ T cell contributed to induce tumor angiogenesis and immune escape, and the activation of the antigen-presenting molecular function participated in melanoma metastasis.

**Conclusion:**

This article provided new insights into underlining VuM molecular regulation and potential signaling involved in immunotherapy, which would benefit the clinical practice and administration.

**Supplementary Information:**

The online version contains supplementary material available at 10.1186/s12885-024-11839-0.

## Introduction

Melanoma originates from pigment-producing melanocytes, which can be found in the skin, eyes, inner ear and soft brain membranes. Genetic variants have been identified as a contributor in such a process. As a kind of malignant tumor, cutaneous malignant melanoma is considered the most aggressive (prone to spread) and fatal skin cancer with a collapsed prognosis which reveals a high possibility of metastasis, although it only accounts for 1% of all skin malignancies. Currently, an integrative therapeutic strategy has been applied in the administration of tumor, including surgical removal, chemotherapy, radiotherapy, photodynamic therapy, immunotherapy or cell therapy [1]. Immunotherapy presents great potential in the advanced management of malignant tumors, but the immune microenvironment is critical in deciding the outcomes of immunotherapy. Thus, beyond histological classification, immunological analysis would be essential for melanoma.

Based on histological analysis, VuM is predominantly superficially diffuse, nodular and acral freckle type, while VaM is predominantly nodular [2, 3]. Superficially diffuse melanoma demonstrates a relatively better prognosis, whereas nodular melanoma indicates a poor prognosis [2]. Generally, VuM and VaM are often subject to delayed diagnoses, and most cases remain undetected until they have progressed to an advanced stage. Female genital tract associated melanoma only consists of 1–3% affected population, and most of them are located in vulva area (76%) [4]. The five-year survival rate of VuM and VaM varies between 10% and 50% [5]. Moreover, a high ratio of local and distant metastases is usually identified in VuM patients, with a high incidence of drug resistance, indicating a terrible prognosis [2, 6].

scRNA-seq has its unique advantage of maximizing tissue heterogeneity owing to unbiased assessment of single-cell expression profile [7]. Since 2009, scRNA-seq has been widely introduced to decompose the heterogeneity of massive tissues [8]. Importantly, the scRNA-seq has been applied in exploring the molecular mechanisms of tumor formation and metastasis, and revealed the tumor microenvironment changes before and after treatment [9, 10]. Also, the functions of non-tumor cells also could be underlined by scRNA-seq, revealing the cellular and molecular atlas of involved cells. Thus, scRNA-seq would benefit to address the cellular and genetic expression heterogeneities in melanoma, which provide advanced information on particular immune microenvironments. Until now, only several uveal melanomas (UVM) had been studied by scRNA-seq, and specific cell-cell communication and immunological regulation signaling had been identified in UVM. However, due to the extremely low incidence of VuM and VaM, there was no available research of such rare melanomas in single-cell resolution. Herein, we reported a rare case of VuM who underwent a surgical removal, and scRNA-seq had been involved to underline the transcriptional profile among various cell types and demonstrate the most highly participated immune cell in regulating the immunological microenvironment.

## Materials and methods

### Ethics approval and consent to participate

The study protocol was approved by the Ethics Committee of the Second West China Hospital of Sichuan University (2014-034). Written informed consent was obtained from the patients from whom the samples were collected. The sample of VuM melanoma (nodular type) was collected by surgical removal from a 68-year-old female patient.

### Isolation of single cells and RNA sequencing

Briefly, cell dispersion was conducted through a tumor dissociation kit produced by Miltenyi Biotec (catlog# 130-095-929). Tumor tissues can be dissociated into single-cell suspensions by combining mechanical dissociation with enzymatic degradation of the extracellular matrix, which maintained the structural integrity of tissues. The tumor tissue was enzymatically digested using the kit components and the gentleMACS™ Dissociators were used for the mechanical dissociation steps. After dissociation, the sample was applied to a filter to remove any remaining larger particles larger than 10,000 bp from the single-cell suspension. The single-cell isolation and labeling platform was based on the ChromiumTM system with Chromium Next GEM Single Cell 3ʹ Library Kit v3.1 (catlog# PN-1,000,157), which simply concentrated cells to 700 ~ 1200 cells/ul (viability ≥ 85%) and loaded approximately 8000 cells per lane to generate single-cell gel beads (bead-in-emulsions, GEMs). After the reverse transcription (RT) step, the GEMs were disrupted and then the Barcoded-cDNA was purified with Dynabeads, followed by PCR amplified. Amplified cDNA was then used for 3’gene expression library construction. For gene expression library construction, 50 ng of amplified cDNA was fragmented and end-repaired, double-size selected with SPRIselect beads, and sequenced on a NovaSeq platform (Illumia) to generate 150 bp paired-end Reads.

### RNA-seq data alignment

The raw fastq data was aligned to the GRCh30-2020-A reference transcriptome provided by 10X genomics (https://cf.10xgenomics.com/supp/cell-exp/refdata-gex-GRCh38-2020-A.tar.gz). The alignment was performed by cellranger v7.0.1 with all default parameters.

### RNA-seq data quantification

We used the percentage of mitochondrial reads as an indicator of quality control. Cells with greater than 1000 genes detected and greater than 1500 unique molecular identifier (UMI) counts and less than 10% mitochondrial gene percentages contained were regarded as qualified cells for downstream analysis. After quality control, 7,257 of the 12,243 cells were retained.

### Data normalizing and cell clustering

Most of the data normalizing and cell clustering steps were performed by R package Seurat v4.0.2. After quality control, the UMI count expression matrix was normalized by Seurat:: NormalizeData( normalization.method="LogNormalize”, scale.factor = 10,000 ). This function will calculate the TPM (Transcripts Per Kilobase of exon model per Million mapped reads) and use the log2(TPM + 1) as a normalized expression value. Then, we ran the Seurat::ScaleData() to scale all normalized expression. The scaled data was mainly used for drawing heatmap. The top 2000 highly variable genes (HVGs) were identified by Seurat::FindVariableFeatures() and used for subsequent Principal component analysis (PCA). Then, the top 15 PCA dimensions were used as input of the Uniform Manifold Approximation and Projection (UMAP) and neighbor matrix calculate and clustering. At last, using the Seurat::FindClusters(resolution = 0.3) to divide cells into clusters.

### Differential expression analysis

Differential expression analysis were performed by Seurat::FindAllMakrer(only.pos = T). The function uses Wilcoxon rank sum test algorithm to identify marker genes which log2(FoldChange) > 0.25 and p-adjusted < 0.05 and min.pct > 0.1.

### InferCNV analysis

InferCNV v1.6.0 was used to explore tumor single-cell RNA-seq data and analyzed them for large-scale chromosome copy number variations (CNV). Since we have only one tumor sample, the inferCNV was run in no-reference mode, i.e., the average expression level of all cells was used as a reference. The cutoff parameter was set to 0.1, which is best for 10X genomic data. Analysis_mode was set to “subclusters”.

### Cell communication

Cell communication analysis was based on the “CellChat” function package, a toolkit for inferring intercellular communication networks and internal regulatory signals by integrating intracellular and intercellular signals. CellChat simulates the probability of cell-cell communication by combining gene expression with previously known knowledge of the interactions between signaling ligands, receptors and their cognates, using a large number of action laws.

### Gene enrichment analysis

Functional enrichment analysis was performed using Gene Ontology (GO, http://www.geneontology.org) and Kyoto Encyclopedia of Genes and Genomes (KEGG, https://www.kegg.jp) analysis for each enriched ontology level (False discovery rate (FDR) < 0.05). For each GO (KEGG ditto), successively counted: (1) Counting the frequency of each GO term occurrence on the whole genome, defined as m; (2) count the number of genes on the whole genome, defined as n; (3) count the number of submitted differential genes in this GO analysis, defined as k; (4) count the frequency of GO in k up-regulated differential genes, defined as o. In R language, the following code “p[i,1] = phyper(o-1, k, n-k, m, lower.tail = FALSE)” could be used to implement the calculation of p-value. Copyright permission of KEGG had been obtained from Kanehisa laboratories [11–13].

## Result

### Case presentation and sample collection

The patient is a 68-year-old female who complained of a duration of 9 months after a perineal lesion was discovered. At the beginning, the patient palpable a perineal mass, accompanied by tenderness, without bleeding, pus, or increased vaginal discharge. Recently, the patient presented difficulty in urinating. Physical examination demonstrated an irregular black lesion located in the inner side of the labia minora and around the urethral opening. A tough mass with a diameter of 3 cm was palpable in the lower part of the anterior vaginal wall, clitoris, and around the urethral opening. Atrophic cervix and uterine body had been identified. No abnormality had been found in appendix palpable. She was diagnosed hypertension and diabetes 2 years ago. Nifedipine and Irbesartan were used to maintain blood pressure, while vildagliptin and gliclazide were administrated for diabetes. The monitor of blood pressure and glucose was recorded as normal in the most recent half year. She stated a regular menstruum, and menopausal occurred at 47 years old.

**Fig. 1:**
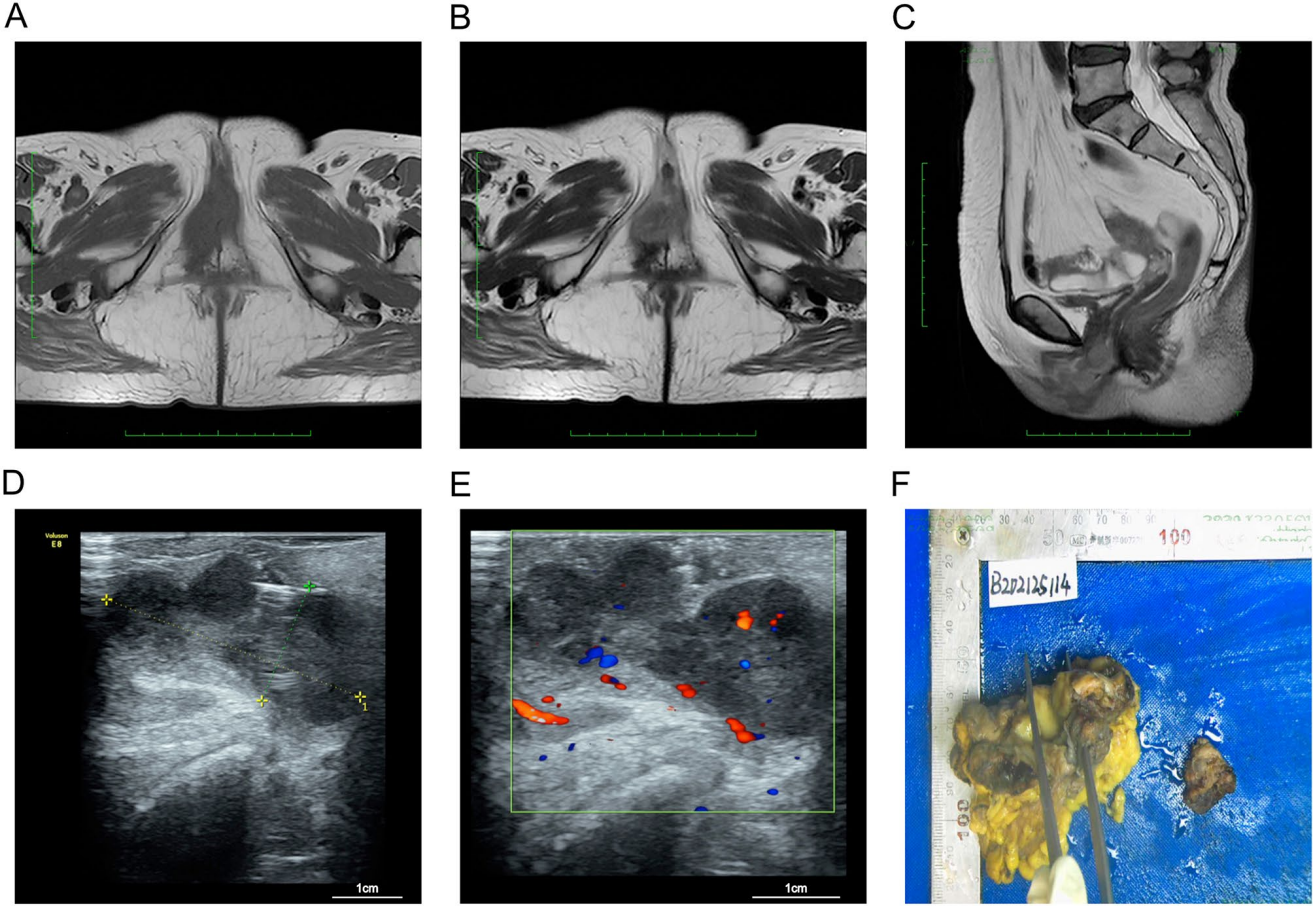
Clinical images of the patient. (A) T1 MRI transverse scanning images of vulvar melanoma in the patient; (**B**) T2 MRI transverse scanning images of vulvar melanoma in the patient; (**C**) T1 MRI longitudinal scanning images of vulvar melanoma in the patient; (**D**) Ultrasound image of vulvar melanoma in the patient; (**E**) Doppler images of enriched blood supplements in vulvar melanoma; (**F**) Sample of vulvar melanoma after surgical removal

Enhanced pelvic MRI (Fig. 1A–C) demonstrated a vulvar mass involving labia and corpus cavernosum, the urethra and the lower part of the vagina; enlarged lymph nodes were detected in the bilateral groin area, left external iliac vessels, and abdominal aorta; multiple small lymph nodes were also detected in the bilateral external iliac vessels and obturator area; while multiple nodules and flaky shadows had been recorded in the pelvic bones, lumbosacral vertebrae, and bilateral femurs, considering bone metastases. Gynecological color doppler ultrasound indicated that the right side of the vulva was detected and found a solid weak echo signaling of 4.0 × 1.7 × 2.5 cm (Fig. 1D and E). Then, the biopsy of the external urethra showed spindle cell tumors, which tended to be sarcoma. Finally, the immunohistochemical diagnosis indicated a malignant melanoma. The patient declined any history of other cancers or tumors, especially for any cutaneous lesions. Moreover, there was no positive familial history of melanoma. According to the clinical presentation and histological examination, the patient was considered as VuM.

After that, a surgical procedure had been scheduled, including extended vulva excision, partial anterior vaginectomy, vulvar plastic surgery, total urethrectomy, bladder neck reconstruction, and cystostomy. Duration surgery, a tumor tissue with a diameter of 3 cm was removed, and the Breslow thickness was estimated as 1 cm (Fig. 1F). The post-surgical histological analysis revealed invasion of the dermal reticular layer (Clark grade IV), and involvement of the urethra and the right medial cutaneous margin of the vulva, without vulvar flap involvement. No further chemotherapy or radiotherapy had been applied for this patient, and the patient has been living for two years after surgery, as follow-up confirmed.

### Identification of cellular and molecular landscape of VuM

**Fig. 2:**
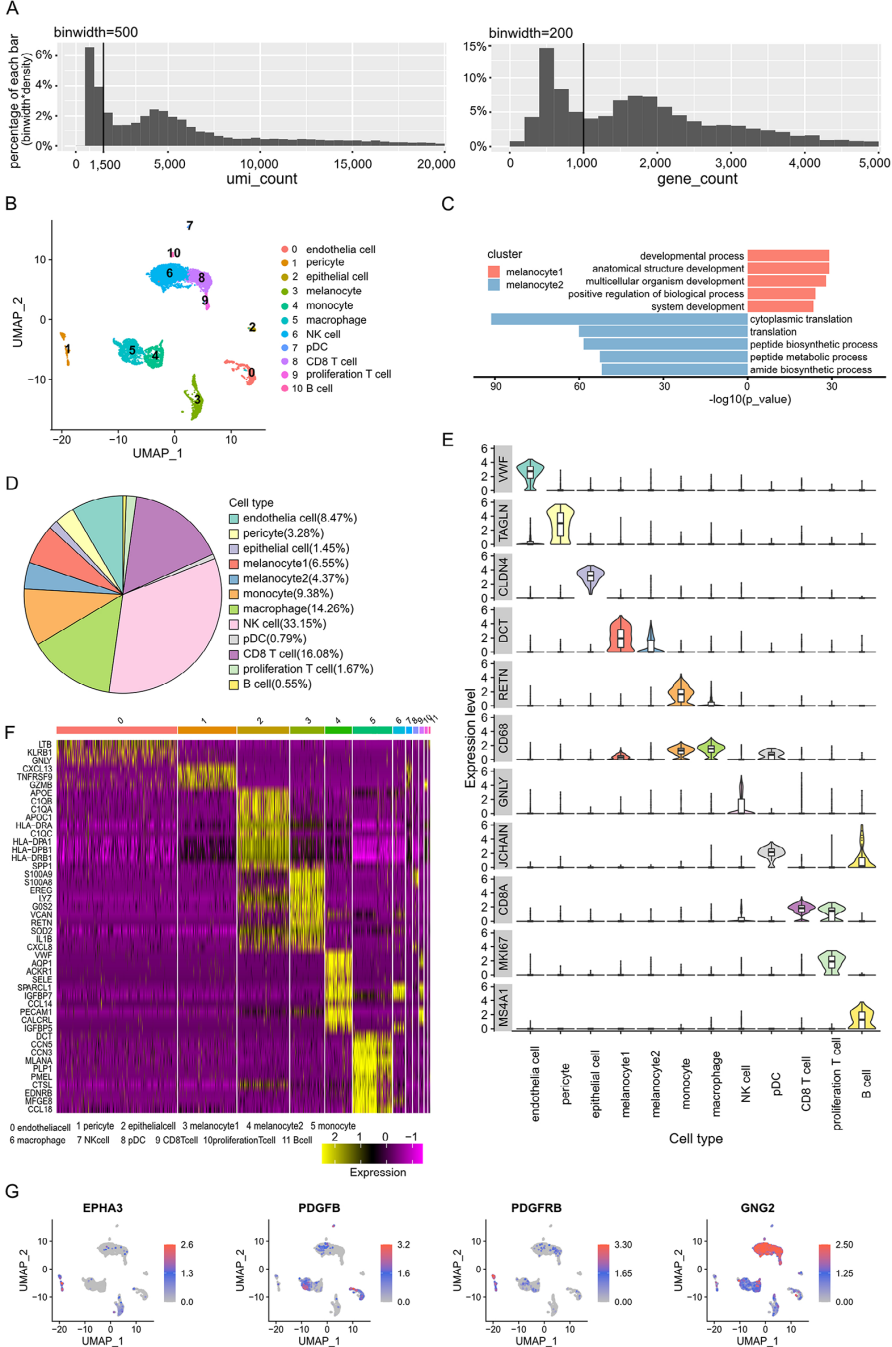
Defining the cellular landscape of vulvar melanoma. (**A**) Distribution of UMI counts and gene counts before quality control. The vertical black lines indicating the QC filter criteria. (**B**) Uniform Manifold Approximation and Projection (UMAP) map showing the identified 12 cell clusters based on the transcriptomes of 12,243 cells. Cells are colored by Seurat clustering and annotated by cell types (each point represents a single-cell). (**C**) Process of subdivided melanocytes into two populations. (**D**). Proportion of cells from major cell types. (**E**) Violin plots showing markers in each cell cluster. (**F**) Heatmap presenting markers in all of the cell types. (**G**) Dotplots depicting the distribution of specific genes in cell landscape, namely EPHA3, PDGFB, PDGFRB, GNG2

**Fig. 3:**
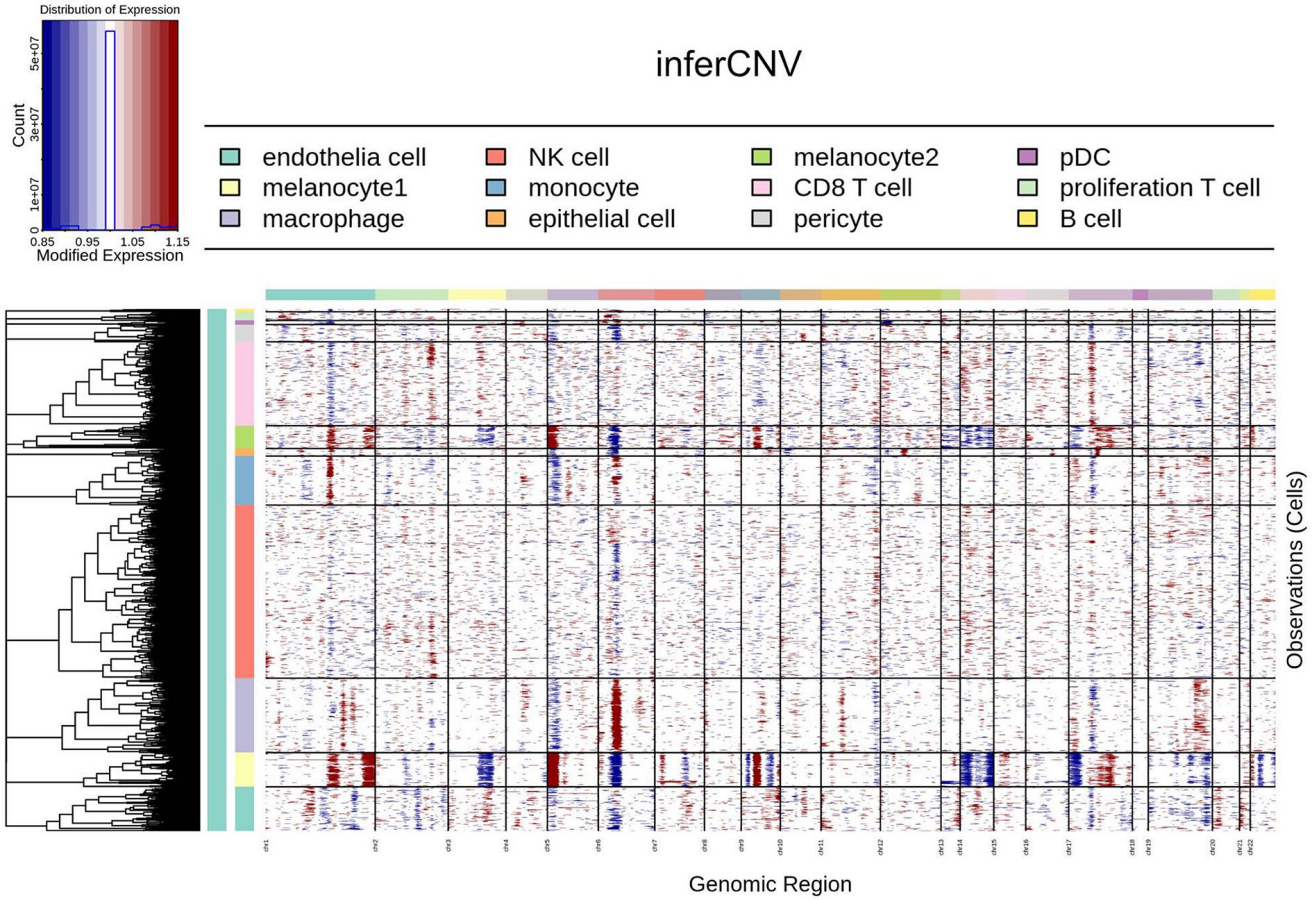
Single cell copy-number variation analysis of vulvar melanoma. The horizontal direction in the figure represents the chromatin position, from chr1 to chr22. The vertical direction represents the cell types. From top to bottom they are B cell, proliferation T cell, pDC, pericyte, CD8^+^ T cell, melanocyte2, epithelial cell, monocyte, NK cell, macrophage, melanocyte1, endothelia cell

scRNA-seq was performed on the VuM. 12,243 transcriptomes were collected after sequencing. Cells with low UMI counts or gene numbers usually indicate that the cells may be cellular debris rather than intact cell. Cells with high mitochondrial transcripts proportion usually indicate that the cells are apoptotic cells via the mitochondrial pathway. So cells with UMI counts lower than 150 or a number of detected genes lower than 1000 or mitochondrial transcripts proportion higher than 10% were regarded as low-quality cells and were removed from downstream analysis (Fig. 2A). After quality control, 7257 cells remained for subsequent analysis. Uniform manifold approximation and projection (UMAP) analysis was performed to investigate the cellular component diversity in the study. Based on the global expression patterns, all cells were classified into 11 major groups, namely B cells, CD8^+^ T cells, endothelial cells, epithelial cells, macrophages, melanocytes, monocytes, NK cells, plasmacytoid dendritic cells (pDCs), pericytes, and proliferation T cells (Fig. 2B). GO results can validate that melanocytes are segregated into two functionally distinct subtypes (Fig. 2C), so we will use 12 cell types for next analysis. Notably, one of these sub-populations, named melanocyte1, exhibited amplification and deletion patterns characteristic of malignant cells, as revealed by InferCNV analysis (Fig. 3).

The transcriptome expression levels of tumor samples can provide insights into the immune activity status of the samples, offering a better understanding into the potential tumor-promoting effects exerted by immune cells. Tumors exhibit high heterogeneity, with variations in immune activity observed among different individuals. However, samples displaying similar immune activity profiles are often found within the same immune microenvironment [14, 15]. Previous studies focusing on melanoma have highlighted the prognostic significance of certain immune cell populations [16, 17]. Specifically, a high density of activated T cells in the peritumoral region, increased levels of B lymphocytes, and mature dendritic cells (DCs), have been associated with longer survival. Conversely, intense infiltration of pDCs or neutrophil granulocytes has been linked to poor prognosis [15, 18]. Furthermore, an elevated ratio of other immune cell types such as macrophages [19], natural killer (NK) cells [20], and monocytes [21] has been implicated in the progression of tumorigenesis, as depicted in Fig. 2D and E.

Classical markers were specifically detected in the corresponding clusters of cells, shown in the heatmap (Fig. 2F). In total, 1,672 differentially expressed genes (DEGs) were obtained among these major cell types using Seurat analysis. We found that four genes (*EPHA3*, *PDGFB*, *PDGFRB*, and *GNG2*) are implicated in cancer cell migration, invasion, and angiogenesis. The expression profiling of these four genes is shown in Fig. 2G.

### InferCNV analysis to determine the malignant cell

As we mentioned above, melanocytes are determined to be the cancerous cell populations in melanoma. So, it was important to explore the mutant cells and the association between genetic variants and tumorigenesis. Using scRNA-seq, it was able to dissect essential gene features based on a multi-step transcriptome analysis to estimate CNVs from averaged gene expression within large chromosomal regions in individual cells (Fig. 3). Hidden Markov and Bayesian latent mixture modeling were performed to determine subclonal CNV events and remove low-confidence CNV cells.

InferCNV analysis of 7257 single cells revealed most of the cell types can be detected CNV in chr6, a result found in most of inferCNV results containing macrophages and B cells, probably due to the massive expression of specific genes in macrophages and B cells. And InferCNV analysis revealed unique chromosomal gene expression patterns in two major cancerous cell populations. Melanocyte1 and melanocyte2 subgroups, which were clearly distinguished from the other cells for the copy number variation, melanocyte1 and melanocyte2 could be found copy number increase in the end of chr1 and head of chr5. Also, copy number decrease could be found in chr3 and chr14 in melanocytes, especially in melanocyte1, which demonstrated the genetic variants could be the original causes of melanoma.

### Enriched gene analysis and cell-cell communication analysis

KEGG (Supplementary Fig. 1) and GO (Supplementary Fig. 2) analysis help to present that each type of cell specifically expressed genes involved in the corresponding biological processes. According to the scRNA-seq analysis, there were two clusters of melanocytes that had been identified, indicating two separated stages in melanocytes differentiation. The cluster melanocyte1 demonstrated significant enrichments of mitochondrial function, including oxidative phosphorylation, cellular respiration, aerobic respiration, and electron transport. Also, this melanocyte1 cluster revealed a highly elevated enrichment of the cell cycle. However, the melanocyte2 presented enrichments among epithelial migration, cell-cell junction, cell-substrate junction, and extracellular matrix (ECM) organization, which indicated that the cluster melanocyte2 still maintains some gene profile features of epithelial cells. The disorders of interactions between epithelial cells and ECM were the main biological processes in the transformation of tumor cells in melanoma. The transformed epithelial cells were recorded with dysfunction in the regulation of normal cell-cell junction and cell-substrate adhesion. While the melanocyte1 cluster should be composed by the mature tumor cells, which presented a high-level mitochondrial function, consisting of the enriched biological process of cell cycle. Taken together, the scRNA-seq revealed two significant separated phases of melanoma formation. The first phase was considered as the transformation from epithelium to unmatured melanocytes, and the second phase was identified as the end-stage differentiation of melanocytes which presented a huge demand of energy and an elevated capability of proliferation.

Besides, in the analysis of KEGG enrichments, Rap1 signaling was activated in T cells, which was one of the RAS family members mediating cell adhesion, cell-cell junction formation, and cell polarity via regulating integrins and other adhesion molecules in various cell types [22]. ATP synthesis coupled electron transport and mitochondrial ATP synthesis coupled electron transport pathways were also up-enriched among melanocytes, pericytes,and T cells. For instance, cyclin-dependent kinase inhibitor 2a (*CDKN2A*, highly expressed in CD8^+^ T cell), located on chromosome 9p21, was associated with familial melanoma syndrome. Mutations of cyclin-dependent kinase 4 (*CDK4*, highly expressed in melanocyte1) had also been shown to predispose to melanoma and represent another checkpoint that may be associated with the development of resistance to BRAF-targeted therapies. What’s more, heat shock protein 90 (HSP90) cooperates with its co-chaperone cell division cycle 37 (*CDC37*, highly expressed in endothelial cells) to support multiple protein kinases involved in cancer progression, including BRAF. As for activated pathways in other T cells, proteasomes were strongly expressed in CD8^+^ T cells which has been already proven as a marker more of nonspecific inflammation than of early cancer [23]. In addition, B cells presented a high level of T cell receptor signaling pathway to assist the regulation of T cells. All the results mentioned above indicated that the activation and invasion of immune cells were critical in melanoma formation and metastasis. So, it was necessary to illustrate the communications and interactions among various immune cells and melanocytes, as well as the connection between stromal cells and melanocytes.

**Fig. 4:**
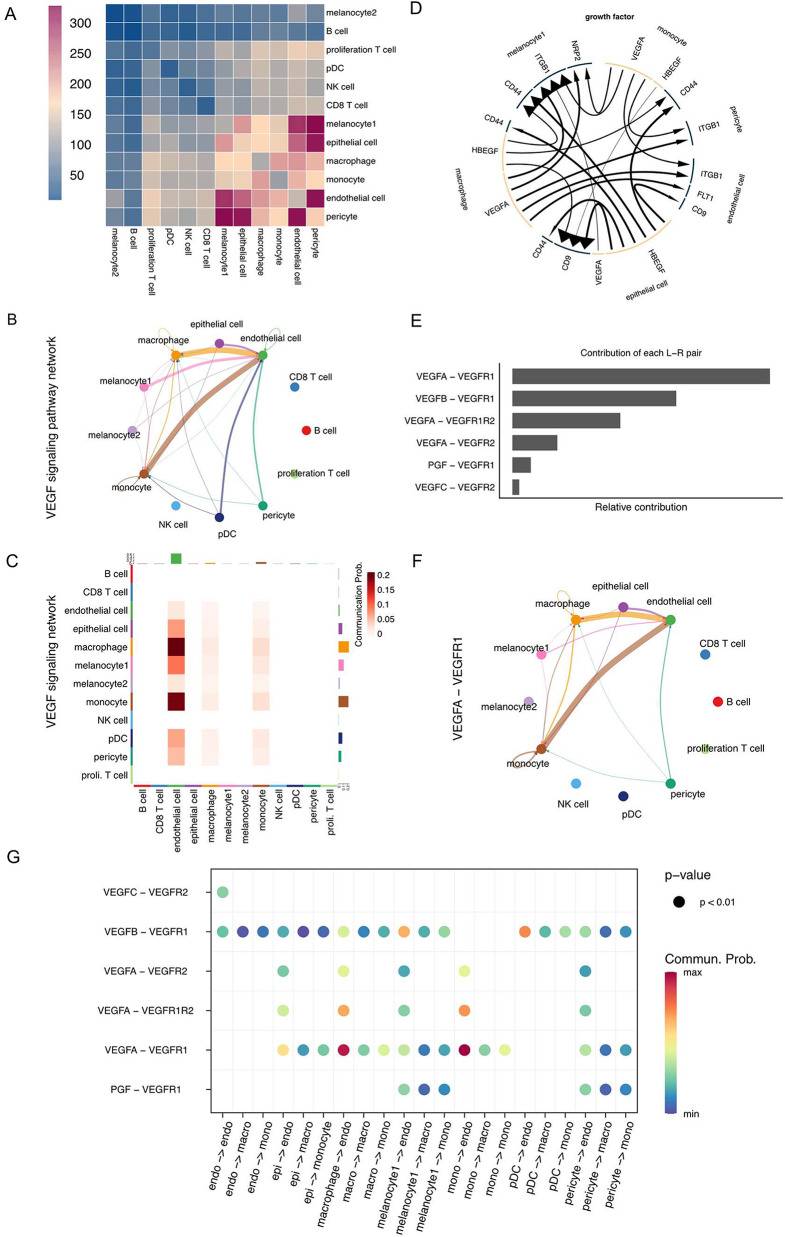
Cell–cell interactions in vulvar melanoma. (**A**) Heatmap displaying intense of cell-cell communication; (**B**) Circle plot for VEGF signaling pathway; (**C**). Heatmap for VEGF signaling pathway; (**D**). Factor receptor ligand chord diagrams. (**E**) Contribution of each ligand pair to VEGF signaling pathway; (**F**). Circle plot for VEGFA-VEGFR1 pair; (**G**). The expression levels of each receptor pair in the VEGF signaling pathway

To investigate the cell-cell communication between these cellular subpopulations, we utilized CellChat analysis to examine the expression levels of ligand-receptor interactions across 12 cell types. We discovered a higher level of interaction pairs expressed between endothelial cells, epithelial cells, monocytes, macrophages, and melanocyte subpopulation 1 (Fig. 4A). Particularly, we identified a strong intercellular VEGF signaling within these interactions (Fig. 4B and C). Furthermore, there was evidence of monocytes regulating genes in other cells, such as *VEGFA*, which can modulate Integrin beta-1 (ITGB1) and cluster of differentiation 44 (CD44) in melanocyte 1, pericyte, and endothelial cells (Fig. 4D). Previous studies have demonstrated that upregulation of ITGB1 and CD44 can promote tumor progression and invasion [24, 25]. The VEGF (vascular endothelial growth factor) pathway plays a crucial role in the process of angiogenesis. Upon activation by VEGF receptors, such as VEGFR-1, VEGFR-2, and VEGFR-3 (Fig. 4E), VEGF, is predominantly produced by tumor cells and other cell types (Fig. 4F and G). Previous studies have demonstrated that *EPHA3* can interact with vascular endothelial growth factor (VEGF) and its receptor VEGFR2, enhancing VEGF-induced angiogenesis in vascular remodeling [26].

## Discussion

VuM and VaM, which represent distinct subgroups of malignant melanoma, exhibit variations in incidence, pathologic features, and clinical outcomes when compared to other subtypes of malignant melanoma [5]. They also present notable biological and therapeutic differences [2]. A study conducted in the United States involving 1863 patients revealed that the American Joint Committee on Cancer (AJCC) staging system for cutaneous melanoma can be applied to VuM and can effectively predict prognostic outcomes based on surveillance and epidemiological findings [2]. However, there is currently no corresponding staging system available for VaM. The literature on VaM is relatively scarce and primarily consists of case series studies [27–29], with insufficient data to determine whether VaM and VuM share similar prognoses. Furthermore, there is no universally recommended staging system specifically designed for VaM, although staging systems such as the International Federation of Gynecology and Obstetrics (FIGO) staging for vaginal cancer and the AJCC staging for melanoma can be referenced. For example, Breslow thickness, an important parameter in the TNM staging system, lacks sufficient clinical data to establish a significant relationship with VaM prognosis, leading to its exclusion from the AJCC staging system for VaM. Moreover, tumor size and lymphatic metastasis, both considered as potential prognostic factors for VaM, are not adequately accounted for in the FIGO staging system for vaginal cancer [29].

By characterizing the transcriptional signatures of various cell types through the isolation and profiling of single cells from VuM, we have provided a comprehensive depiction of the genetic landscape encompassing 12 distinct cell subtypes. Within our VuM sample, we observed a substantial presence of immune infiltrates and a minority population of cancer cells primarily comprising melanocytes.

Among the identified markers associated with melanoma, *EPHA3*, encoding the EPH receptor, has been implicated in cell repulsion, adhesion, and motility. The EPH receptor functions as a tumor formation inhibitor, and the depletion or mutation of the *EPHA3* gene reduces its suppressive effect on tumor proliferation, thereby promoting tumor metastasis [30]. Previous studies have also demonstrated that the activation of *EPHA3* is observed in certain melanoma samples, leading to tumor metastasis through the modulation of Rho-dependent cytoskeleton reorganization and cell retraction [31, 32]. Notably, Fig. 2G reveals the overexpression of *EPHA3* in pericytes, which are involved in the functional regulation of blood vessels and angiogenesis through interactions with endothelial cells [33]. Intriguingly, our findings indicate a propensity for pericytes to transform into CAFs, mediated by the activation of *MRC2* [34] and co-expression of genes such as *Col15a1*, *Pi16*, *Dpt*, *CCL19*, and *COL3A1* [35]. CAFs play a pivotal role in suppressing immune cells through the secretion of various cytokines and metabolites, thereby facilitating cancer progression, invasion, and metastasis [36]. Existing studies have highlighted the capacity of epithelial cells, endothelial cells, bone marrow mesenchymal stem cells (MSCs), and other cell types within tumor tissues to differentiate into CAFs [37, 38]. Consequently, it is crucial to preserve pericyte gene expression and prevent their transformation into CAFs within the tumor microenvironment. Moreover, our scRNA-seq analysis revealed a significantly elevated expression of PDGFB signaling. In vitro studies have shown that PDGFRβ induces the phosphorylation of EPHB4 upon stimulation by its ligand, PDGF-BB. Subsequently, the PDGF-BB ligand activates PDGFRβ and EPHB4 through direct and indirect mechanisms, leading to the downregulation of Akt and Erk1/2 pathways, which enhance tumor cell survival and proliferation [39, 40]. It was showen that *EPHA3* had a high expression at pericyte. While *GNG2* was significantly highly expressed in the NK cell and CD8^+^ T cell. And as *PDGFB* and its receptors PDGFRB’s expression profiles were relatively consistent, but with a different expression level. For example, Pericytes had high *PDGFRB* expression but low *PDGFB* expression, and macrophages had low *PDGFRB* expression and high *PDGFB* expression.

This dysregulated immune microenvironment characterized by abnormal PDGFB signaling and the presence of CAFs is likely responsible for the unfavorable prognosis and suboptimal response to immunotherapy observed in melanoma. It underscores the importance of understanding the intricate interplay between immune cells, pericytes, and other cellular components within the tumor microenvironment. Maintaining the expression of pericyte-specific genes while preventing their transformation into CAFs is crucial for effective tumor microenvironment management. Strategies aimed at modulating the PDGFB signaling pathway and targeting EPH receptors could offer potential therapeutic avenues for controlling tumor cell survival, proliferation, and metastasis. Furthermore, the identification of EPHA3 as a significant player in melanoma progression suggests its potential as a therapeutic target. Inhibition of EPHA3 activity could potentially impede tumor metastasis by preventing cytoskeleton reorganization and cell retraction mediated by its downstream signaling pathways. Overall, our findings shed light on the complex molecular interactions and cellular dynamics within the melanoma microenvironment. They highlight the potential of targeting EPHA3, PDGFB signaling, and the modulation of pericyte-CAFs transition as promising strategies for developing novel therapeutic interventions and improving patient outcomes in melanoma. Further investigations are warranted to elucidate the precise mechanisms underlying these processes and validate their clinical relevance.

The formation of cell-ECM interactions plays a crucial role in cell-cell and cell-substrate connections, as revealed by the analysis of GO and KEGG terms. Targeting these cancer ECM interactions and tumor mechanisms holds promise for improving the efficacy of targeted therapy in melanoma. The ECM is a dynamic network of macromolecules with distinct biochemical and mechanical properties, contributing to the establishment of the tumor microenvironment. Aberrant ECM deposition, fibrous alignment, and collagen cross-linking lead to tumor sclerosis, which correlates with increased cancer risk and unfavorable clinical outcomes in breast and pancreatic cancer patients [41]. The challenges in eradicating melanoma stem from its inherent heterogeneity and adaptability. Melanoma exhibits significant heterogeneity in terms of cell composition, chromosomal structure, developmental trajectories, intercellular signaling networks, and phenotypic dominance. By analyzing metabolic gene expression profiles in the tumor microenvironment using single-cell expression data, researchers can uncover metabolic characteristics and pathways that distinguish tumor metabolic heterogeneity at the single-cell level from that at the tissue level. This approach enables the determination of tumor microenvironment heterogeneity by re-subtyping and identifying cellular features, including phenotypic abundance, genetic alterations, immune dynamics, clonal expansion, developmental trajectories, and molecular interactions [42]. These factors have the potential to influence patient prognosis and treatment outcomes, providing invaluable insights into melanoma treatment strategies [43]. A significant portion (40-50%) of malignant melanoma arises from immune-related antigens, such as T cell-granulocyte expression of PD-1, CD8^+^, CD4^+^ T, and NK T lymphocyte-granulocytes. Combining PD-1/PD-L1 inhibitors with Toll-like receptor agonists has been extensively studied as a combination therapy in clinical practice. Immuno-infiltration therapy aims to enhance the immune response against tumor-associated antigens or autoantibodies. While T lymphocytes can directly eliminate tumor cells, it is crucial to disrupt the balance of CD8^+^ T and NK lymphocytes-granulocytes within the tumor to prevent malignant melanoma metastasis recurrence. This can be achieved by activating CD8^+^ T lymphocytes-granulocytes through antigen recognition receptors or ligands, a process known as anti-demethlicity. Another approach involves obtaining resistant T lymphocytes-granulocytes by adding or removing antigens in immunodeficient mouse allografts. PD-1/PD-L1 inhibitors have demonstrated efficacy in the treatment of non-metastatic melanoma and various other malignancies. Currently, PD-1/PD-L1 inhibitors are approved for the treatment of melanoma, bladder cancer, and other malignancies. Thus, the scRNA-seq revealed the significance of immune responses involved in cellular communication contributing to micro-environment formation in melanoma. And the cellular interaction induced several antigens’ high expression which participated in the immunological escape of cancer cells and the failure of chemical therapy. So, the identified results demonstrated the importance of immunological regulation or modification in melanoma management. Although this was only a case report which contained limited data and samples. However, the results still provided clinical implications but more than 10 thousand cellular interaction analysis, indicating a further research topic hitting the immunological regulation or immunotherapy in melanoma.

There are still many limitations in this study. Firstly, the sample size was relatively small, consisting of only one specimen. Furthermore, when comparing our data to other datasets such as The Cancer Genome Atlas (TCGA) and other single-cell datasets, it is crucial to consider potential confounding factors arising from technical and batch effects, emphasizing the need for cautious interpretation. To address these limitations, we plan to increase the sample size in our follow-up study to ensure the reproducibility of our conclusions. To further validate the critical role of CD8^+^ T cells, we will use PD-1/PD-L1 inhibitors to treat human VuM cells. By scratch assay and transwell invasion assay, we can verify the effect of CD8^+^Tcell on the VuM cells migration. Hoping experiment results provide a new reference for VuM targeting treatment.

## Conclusion

In conclusion, the characterization of melanoma at the cellular and molecular levels has provided valuable insights into its heterogeneity, tumor microenvironment, and potential therapeutic targets. Studies examining single-cell expression data have revealed transcriptional signatures of various cell types, allowing for the identification of specific markers and pathways involved in melanoma progression and metastasis. Besides, the immunological microenvironmental regulation had been supposed to be involved in the formation and growth of melanoma, and corresponding molecules on the identified antigens presented a promising therapeutic strategy. Furthermore, the identification of genetic alterations, metabolic characteristics, and molecular interactions within the tumor microenvironment provides valuable information for personalized treatment approaches and prognostic predictions. l.

### Electronic supplementary material

Below is the link to the electronic supplementary material.

Supplementary Material 1

## Data Availability

The original contributions presented in the study are included in the article; further inquiries can be directed to the corresponding authors. The data that support the findings of this study have been deposited into the CNGB Sequence Archive (CNSA) of China National GeneBank DataBase (CNGBdb) with accession number CNP0004387.
