# Supplementary material for: Single-cell analysis of the cellular landscape of vulvar melanoma provides new insight for immunotherapy administration
Source: BMC Cancer. 2024 Jan 17;24:101. doi: 10.1186/s12885-024-11839-0 (PMC10795381; doi:10.1186/s12885-024-11839-0)
Supplement: Supplementary file 1 — Supplementary Material 1 [file 12885_2024_11839_MOESM1_ESM.docx]

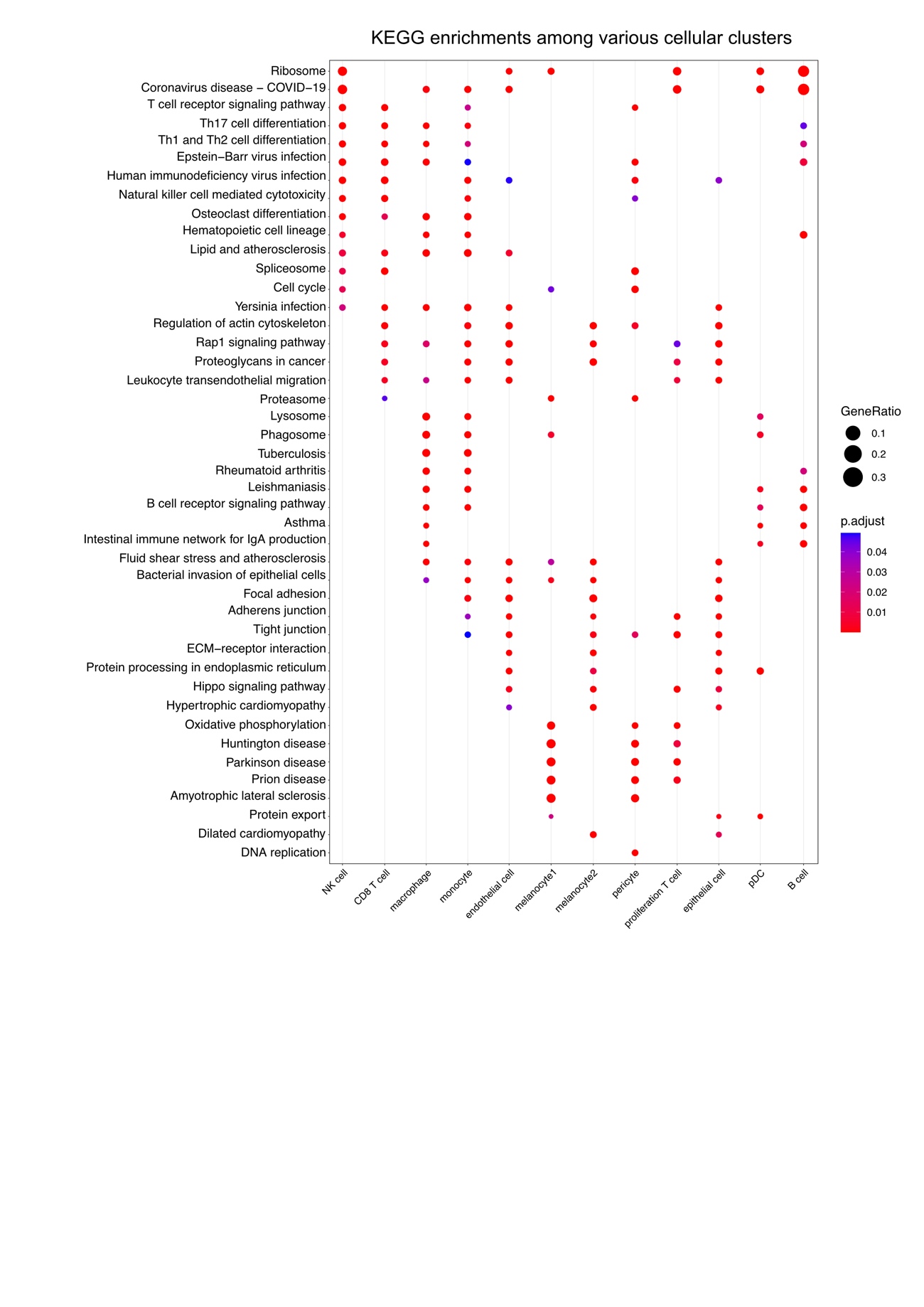


**Supplementary Figure 1. Dotplot presented enriched KEGG pathw­ays.**

**
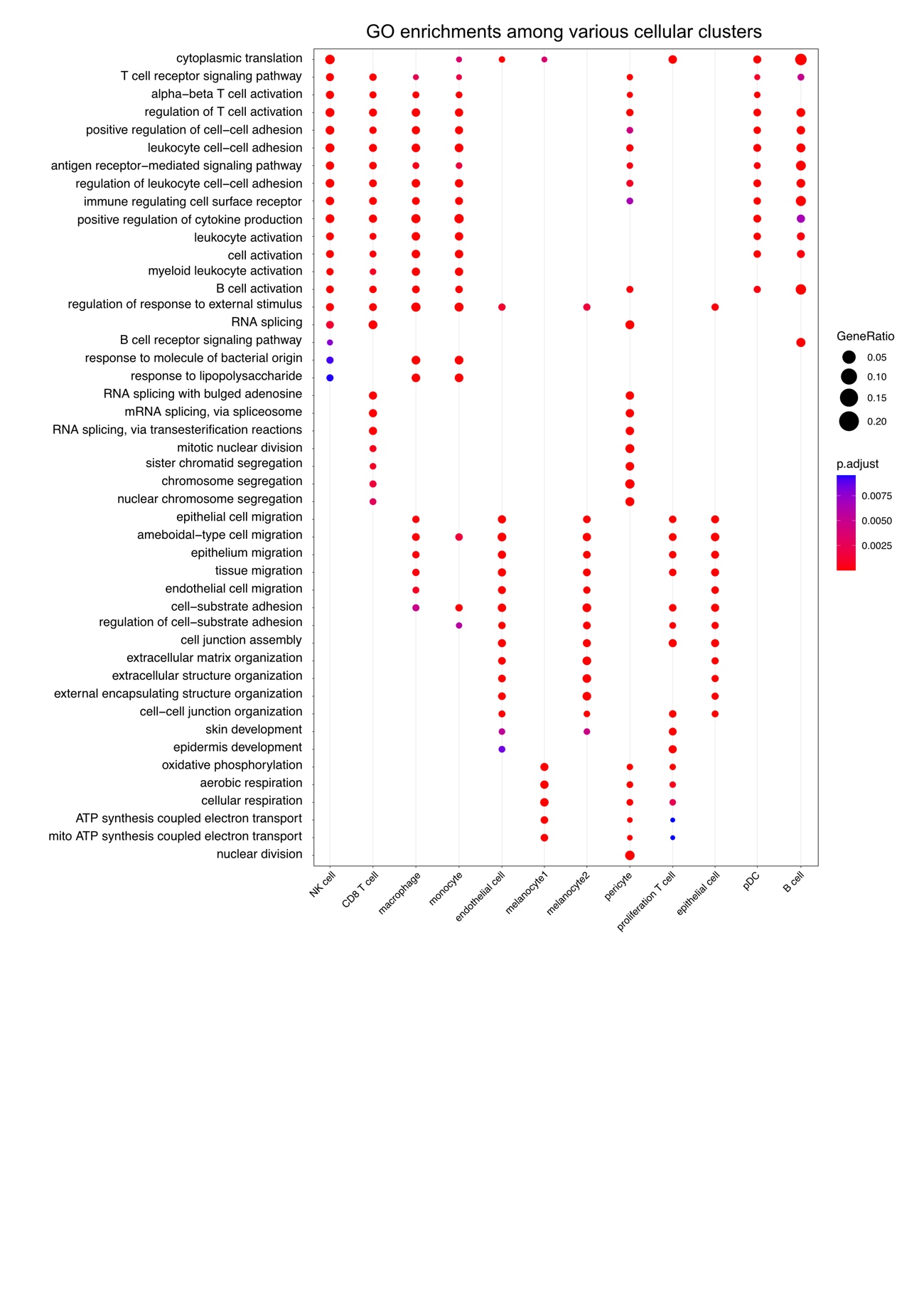
**

**Supplementary Figure 2. Dotplot presented enriched GO terms.**
